# Flaw Detection in Highly Scattering Materials Using a Simple Ultrasonic Sensor Employing Adaptive Template Matching

**DOI:** 10.3390/s22010268

**Published:** 2021-12-30

**Authors:** Biao Wu, Yong Huang

**Affiliations:** 1 College of Civil Engineering, Nanjing Tech University, Nanjing 211816, China; 2 Key Lab of Structures Dynamic Behavior and Control of the Ministry of Education, School of Civil Engineering, Harbin Institute of Technology, Harbin 150090, China; huangyong@hit.edu.cn; 3 Key Lab of Smart Prevention and Mitigation of Civil Engineering Disasters of the Ministry of Industry and Information Technology, Harbin Institute of Technology, Harbin 150090, China

**Keywords:** ultrasonic sensor, nondestructive testing, flaw detection, grain noise, adaptive template matching

## Abstract

Ultrasonic sensors have been extensively used in the nondestructive testing of materials for flaw detection. For polycrystalline materials, however, due to the scattering nature of the material, which results in strong grain noise and attenuation of the ultrasonic signal, accurate detection of flaws is particularly difficult. In this paper, a novel flaw-detection method using a simple ultrasonic sensor is proposed by exploiting time-frequency features of an ultrasonic signal. Since grain scattering mostly happens in the Rayleigh scattering region, it is possible to separate grain-scattered noise from flaw echoes in the frequency domain employing their spectral difference. We start with the spectral modeling of grain noise and flaw echo, and how the two spectra evolve with time is established. Then, a time-adaptive spectrum model for flaw echo is proposed, which serves as a template for the flaw-detection procedure. Next, a specially designed similarity measure is proposed, based on which the similarity between the template spectrum and the spectrum of the signal at each time point is evaluated sequentially, producing a series of matching coefficients termed moving window spectrum similarity (MWSS). The time-delay information of flaws is directly indicated by the peaks of MWSSs. Finally, the performance of the proposed method is validated by both simulated and experimental signals, showing satisfactory accuracy and efficiency.

## 1. Introduction

Ultrasonic sensors have been widely used as a nondestructive testing (NDT) method to detect hidden flaws in materials. The well-known ultrasonic pulse-echo method, first proposed by Pellam and Galt [1], is based on the important property of ultrasound that the ultrasonic pulse signal can be reflected at inhomogeneities and discontinuities in the material, and the reflected signal contains valuable information on the condition of the material or structure. Based on this simple property of ultrasound, great advances have been made both in ultrasonic sensor technology [2,3,4,5] and related NDT methodologies [6,7,8]. Embedded ultrasonic sensors are also widely used as an effective tool for structural health monitoring (SHM) purposes [9,10]. More recently, novel NDT methods based on coda wave interferometry (CWI) have been proposed as a promising technique to detect multiple cracks [11] and to monitor weld fatigue crack growth [12]. However, when using ultrasonic sensors for the inspection of highly scattering materials such as concrete, heat-treated stainless steel, and alloys, the received signals are usually contaminated by strong noise from both the electronic system of the instruments and scattering by the microstructure of the material (also known as the grain) being tested. Due to its origin, the latter is often called grain noise or structural noise. Encountered mostly in the testing of coarse-grain materials, grain noise will contaminate the defect echo in the ultrasonic signal, or even completely submerge it, which greatly limits the effectiveness of defect detection, even with the help of a modern, sophisticated ultrasonic sensor system. Additionally, the spectra of flaw echo and grain noise show significant overlapping, making the elimination or suppression of grain noise a challenging task.

To reduce noise or to enhance the SNR of the signal, several signal-processing techniques have been proposed. One well-known frequency diversity technique is split spectrum processing (SSP) [13,14,15]. SSP first divides the spectrum of the received signal into several sub-bands to which nonlinear processing, such as minimization or polarity thresholding, is applied; they are then transformed back to the time domain, and finally, they are recombined to form the de-noised signal. This processing can be effective for flaw detection if parameters are tuned properly, but it also inevitably produces distorted waveforms, and the performance highly depends on the parameter selection [16]. Approaches such as wavelet transform [17,18,19,20] and S-transform [21,22] are also widely researched. While wavelet analysis possesses many desirable properties by means of analytical functions that are local in both time and frequency, the major drawback of this method is that its performance depends on the selection of the mother wavelet function, the thresholds, the decomposition level of the signal, as well as the order of the mother wavelet function. Recently, signal-processing techniques adopting the idea of sparse signal representation for noise suppression were also developed and show good potential [16,23,24,25]; however, these methods are too complicated and inefficient for practical use.

When an ultrasonic pulse propagates inside a coarse-grain material, the pulse will interact with both grains and flaws, which results in the distortion of the pulse due to scattering and attenuation. As reported in [26], grain sizes $\bar{D}$ often range from 10 μm up to 140 μm. For ultrasonic nondestructive testing, ultrasonic sensors with frequencies from 2 MHz to 10 MHz (wavelengths λ range from 0.5 mm to 3 mm) are commonly seen in practice. Because of the ratio of the wavelength to grain diameter $\lambda /\bar{D}\gg 1$, grain scattering mostly happens in the Rayleigh scattering region, where attenuation is dependent on the fourth power of frequency [26]. An ultrasonic pulse propagating through such a medium will suffer spectral distortion due to the higher rate of attenuation of high-frequency components compared to low-frequency components. On the other hand, the amplitude of grain scattering is proportional to the second power of frequency in the Rayleigh scattering region [27]; therefore, high-frequency components will have larger backscattered intensity compared to low-frequency components. A flaw, though, can be viewed as a geometrical reflector, and thus the reflection is regarded as independent of frequency [28]. As a result, a difference can be found in the interaction of the incident ultrasonic pulse between the grain and flaw, although both the grain-scattered signal, which forms grain noise eventually, and the flaw-reflected echo suffer from frequency-dependent attenuation. This subtle difference, if taken advantage of properly, could be helpful for flaw detection.

On the basis of the above analysis, this paper presents a novel methodology by exploiting the time-frequency feature of ultrasonic signal for flaw detection in highly scattering materials using a conventional normal-incidence longitudinal wave ultrasonic sensor. However, one of the factors limiting effective flaw detection is that the attenuation coefficient of ultrasound in the material is mostly unknown beforehand, and its estimation is not easy in NDT practice. In this study, a new method for estimating the attenuation coefficient is also developed. We start with the spectral modeling of grain noise and flaw echo, and how the two spectra evolve with time is established. Then, a time-adaptive spectrum model for flaw echo is proposed, which serves as a template for the flaw-detection procedure. Next, a sparseness-promoting similarity measure is proposed, based on which the similarity between the template spectrum and the spectrum of the signal at each point is evaluated sequentially, revealing a series of similarity coefficients called the moving window spectrum similarity (MWSS), which only contains a few nonzero entries. The peaks of MWSSs will directly indicate the time delays of the flaw echoes, and the location of flaws can be readily obtained using the time-of-flight principle.

This work mainly focuses on the detection of flaws using a simple ultrasonic sensor working in pulse-echo mode. This fundamental testing scheme is the basis of many advanced ultrasound-based techniques, such as ultrasonic array imaging [5,7]. The rest of this paper is organized as follows. Section 2 is devoted to a detailed analysis of the spectral characteristics of each component of the ultrasonic NDT signal. In Section 3, the proposed methodology is presented in detail. In Section 4 and Section 5, we demonstrate our method by synthetic ultrasonic signals and signals from a laboratory-conducted ultrasonic NDT of a stainless steel block that contains prefabricated flaws, respectively. In the final section, some conclusive remarks are drawn, and a discussion is presented.

## 2. Modeling of the Ultrasonic NDT Signal

Consider a typical ultrasonic NDT scenario conducted in the pulse–echo method, and the specimen being inspected is a block of coarse-grained material. Due to the coarse-grained microstructure of the material, scattering will occur when the out-going ultrasonic pulse from the transmitter impinges on the boundaries of the grains that are randomly distributed in the space domain, generating echoes that seem to be randomly distributed in time. These echoes picked up the receiver are usually referred to as backscattering noise, also known as grain noise or structural noise. Furthermore, the stochastic disturbance in the circuits of instruments of the ultrasonic NDT system also creates a type of noise. Therefore, the measured ultrasonic signal y(t) can be written as y(t)=e(t)+g(t)+w(t), where e(t) refers to the echoes reflected by flaws in the block, and g(t) and w(t) are backscattered grain noise and the electrical white Gaussian noise from the circuits, respectively.

### 2.1. Modeling of Grain Noise

In pulse-echo mode where a transducer is working as both the transmitter and receiver, the scattered wave by a scatterer in the material under inspection in the time domain can be modeled as the convolution of the impulse response of a grain scatterer r(t,λ) and the impinging ultrasonic wave h(t,λ) on the scatterer:

(1) g(t)=h(t,λ)∗r(t,λ)

where ∗ denotes the convolution operator. The impulse response function r(t,λ) gives a mathematical description of the backscattered wave as a function of wavelength λ (or equivalently, frequency) in the time domain. It is also worthwhile noting that impinging ultrasonic pulse h(t,λ) is the convolution of transmitted pulse u(t,λ) and attenuation function a(t,λ), since the transmitted pulse will experience frequency-dependent attenuation during its flight from the transmitter to the scatterer. Therefore, the backscattered echo by a grain scatterer received by the receiver can be expressed as:

(2) g(t)=u(t,λ)∗a(t,λ)∗r(t,λ)

In the frequency domain, the expression of the above equation can be written as:

(3) G(f)∝|A(f)||S(f)||R(f)||U(f)|

where A(f) is the transfer function corresponding to the attenuation characteristics and is expressed as [24]:

(4) $$\begin{array}{cc} A(f) & \propto \exp[-2\int_{0}^{z}\alpha (z,f)dz]\\ & =\exp\{-2\int_{0}^{z}[\alpha_{a}(z,f)+\alpha_{s}(z,f)]dz\} \end{array}$$

with z being the position of grain scatterer. In Equation (4), α(z,f) is the frequency-dependent attenuation coefficient defined as the sum of the scattering coefficient $\alpha_{s}(z,f)$ and the absorption coefficient $\alpha_{a}(z,f)$. In the Rayleigh scattering region, where the wavelength λ is larger than the average grain diameter $\bar{D}$, the scattering coefficients $\alpha_{s}(z,f)$ vary with the third power of the grain diameter and the fourth power of frequency, while the absorption coefficient increases $\alpha_{a}(z,f)$ linearly with frequency [29]. R(f) is the frequency-dependent scattering function. In the Rayleigh scattering region, R(f) is proportional to the second power of frequency; thus, high-frequency components will have larger backscattered intensity compared to low-frequency components. S(f) is the frequency-modulation function due to the sum of grain scatterers with random orientations [30]; it represents the random distribution of phases of grain scatterers. U(f) is the frequency response of the transducer and can be modeled as a Gaussian-shaped spectrum.

### 2.2. Modeling of a Flaw Echo

Similar to grain noise, a flaw echo can also be modeled as a convolution between the impinging ultrasonic pulse and the impulse response function of a flaw, also considering the effect of frequency-dependent attenuation:

(5) $$e(t)=u(t,\lambda )*a(t,\lambda )*p_{f}(t,\lambda )$$

where $p_{f}(t,\lambda )$ is the impulse response function of a flaw. Generally, a flaw can be viewed as a geometrical reflector since its size is larger than the ultrasonic wavelength; thus, the reflection at a flaw is regarded independent of frequency and $p_{f}(t,\lambda )$ has no impact on the frequency-domain expression of a flaw echo. Therefore, the frequency-domain expression of a flaw echo can be written as:

(6) E(f)∝|A(f)||U(f)|

## 3. The Proposed Flaw-Detection Method

The modeling in Section 2 shows that the spectrum of a flaw echo will exhibit a slightly different pattern when compared to the spectrum of grain noise, as can be observed by comparing Equations (3) and (6). The distortion of a flaw echo is mainly caused by frequency-dependent attenuation that high-frequency components attenuate more than low-frequency components; thus, the spectrum of a flaw echo shows a downward shift compared to the frequency spectrum of the transmitted ultrasonic pulse. On the other hand, the spectrum of grain noise will be influenced by frequency-dependent attenuation, frequency-dependent scattering, and the randomness of the orientations of grains. Since high-frequency components have higher scattering strength in the Rayleigh scattering region, the spectrum of grain noise shows an upward shift. Utilizing these features, we propose a novel flaw-detection method as follows.

### 3.1. Estimating the Spectrum of a Flaw Echo

Since frequency-dependent attenuation is the main cause of a distorted flaw echo, and its impact is explicit in the frequency domain, the spectrum information can be almost predicted using the model given by Equation (6). The spectrum of the transmitted pulse can be easily obtained by performing a fast Fourier transform (FFT) on the time domain echo reflected by a plane reflector in the far field of the transducer. Generally, the spectrum of the transmitted pulse can be assumed to have a Gaussian shape, which is given as:

(7) $$U(f)=\exp[-{\left(f-f_{c}\right)}^{2}/2s^{2}]$$

where $f_{c}$ is the center frequency and s is a parameter that controls the frequency bandwidth or the duration of the pulse in the time domain.

In the Rayleigh scattering region, it can be seen from Equation (4) that the attenuation of a pulse during its propagation in the material is due to the combined influence of material scattering and absorption. However, the absorbing term is usually insignificant and negligible when compared to the scattering term [29]; therefore, we only consider the latter in the attenuation model, which can be expressed as follows:

(8) $$A(f,t)=\exp[-\frac{1}{2}\alpha {\left(2\pi f\right)}^{4}vt]$$

where v is the longitudinal velocity of sound in the material being inspected. Equation (8) describes the attenuation of the pulse when it travels a distance d=vt. Apparently, the attenuation term is also a function of time, or equivalently a function of distance. From Equation (8), it is observed that once the attenuation coefficient α is given, the attenuation of a pulse during its propagation can be estimated, using the model given in Equation (6). For example, if a potential flaw echo exists at a time point $t_{i}$ in the measured signal, the spectrum of this flaw echo can be estimated as the product of Equations (7) and (8).

Since the spectrum for a potential flaw at any given time can be predicted, if there is a flaw at $t_{i}$, in the measured signal, then the local spectrum of the signal will exhibit a high degree of similarity to the predicted spectrum. Based on this principle, a novel flaw-detection approach can be developed. First, the predicted spectrum of a flaw echo at a given time point $t_{i}$ is estimated and served as a template. At the same time, the local spectrum of the signal at $t_{i}$, obtained by performing FFT on a narrow window of data, is compared with the template in terms of similarity, revealing a similarity coefficient. By moving the window sequentially along the time axis, a series of similarity coefficients termed MWSS can be generated for the whole signal. The peaks of MWSSs will directly indicate the time delays of the flaw echoes. The location of flaws can be readily obtained using the time-of-flight principle. However, the value of the attenuation coefficient α needs to be determined first.

### 3.2. Estimation of Attenuation Coefficient

It can be seen that the attenuation coefficient α is of vital importance in predicting the spectrum of a flaw echo. Unfortunately, the value of α is not usually available and not easy to measure in practice. In this study, a simple approach for estimating the attenuation coefficient is presented.

Now, suppose a pulse is propagating in the material, and the pulse has a Gaussian-shaped spectrum given in Equation (7). After propagating a distance of d in the material, the frequency spectrum of the pulse can be predicted by Equation (6), given as:

(9) $$\begin{array}{cc} Q_{\left(d\right)}(f) & =\exp[-{\left(f-f_{c}\right)}^{2}/2s^{2}]\cdot \exp[-8\pi^{4}\alpha df^{4}]\\ & =\exp[-\frac{16\pi^{4}\alpha ds^{2}+f^{2}+f_{c}{}^{2}-2f_{c}f}{2s^{2}}] \end{array}$$

By taking the first-order derivative of $Q_{\left(d\right)}(f)$ with respect to f and setting it to zero, the new center frequency $f_{d}$ can be obtained. After some appropriate rearrangements, the following relation can be found:

(10) $$32\pi^{4}\alpha s^{2}df_{d}{}^{3}+f_{d}-f_{c}=0$$

In practice, the echo reflected by the bottom of the specimen can be extracted and used to estimate the attenuation coefficient. The center frequency of the bottom-reflected echo, denoted as $f_{b}$, can be easily obtained by FFT. The distance traveled by the bottom-reflected echo equals two times the thickness of the specimen l. After substituting d=2l into Equation (10), the attenuation coefficient α can be estimated as:

(11) $$\alpha =\frac{f_{c}-f_{b}}{64\pi^{4}s^{2}f_{b}l}$$

### 3.3. Flaw Detection by Adaptive Template Matching

**Figure 1:**
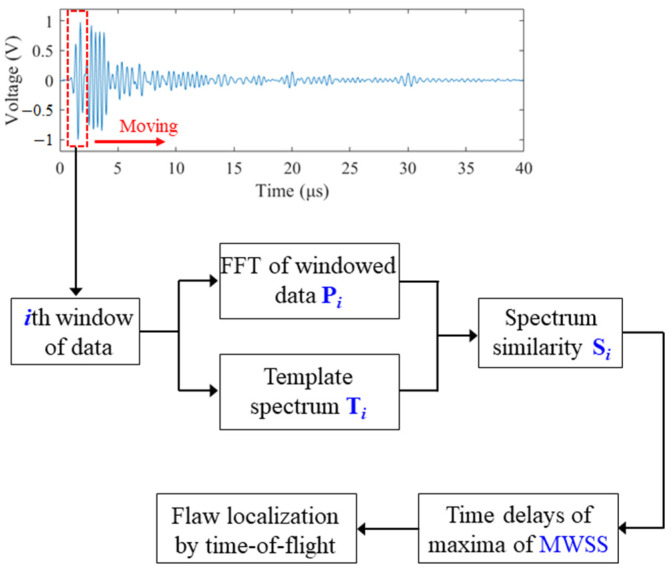
The proposed flaw-detection scheme.

In Figure 1, the proposed procedure is demonstrated. A narrow window of data is first selected, and the FFT operation is performed on the windowed data to extract its spectrum $P_{i}$. The determination of window size depends on the duration of the transmitted pulse, the spatial resolution, and the constraints of spectral analysis techniques. In this study, the widely used Hamming window function is adopted before FFT calculation. The width of the window is set to be slightly larger than the duration of the transmitted pulse. During the propagation of an ultrasonic pulse in the material, due to frequency-dependent attenuation, the frequency bandwidth decreases, and thus the duration of the pulse becomes longer. In practice, the width of the window can be chosen as the duration of the echo reflected by the bottom of the specimen, which is theoretically the longest pulse in the measured signal. This window length can give good spectral estimates without distorting the spectrum.

If the center of the time window is $t_{i}$, the template spectrum $T_{i}$ for this time window can be constructed by combining Equations (7) and (8); that is, $T_{i}=\exp[-{\left(f-f_{c}\right)}^{2}/2s^{2}]\cdot \exp[-\frac{1}{2}\alpha {\left(2\pi f\right)}^{4}vt_{i}]$. Since for each $t_{i}$, the template predicts a spectrum for a potential flaw echo centered at $t_{i}$ by considering frequency-dependent attenuation and the traveled distance $d_{i}=vt_{i}$, it can be seen that this template is adaptive to the signal distortion at any given time $t_{i}$.

It should be noted that testing conditions such as temperature and moisture will influence the sound velocity in the material. For each testing, the measurement of sound velocity in the material should be conducted to consider the influence of environmental conditions. Another important factor that should be considered is the load effect. As has been confirmed in earlier works [9,11], wave velocity is strongly related to the elastic properties of the propagation medium. A linear relationship between wave propagation velocity and elastic strain exists when a limited load is acting on the material. However, when the strain variation exceeds a certain level, the linear relationship is broken. Moreover, temperature variation may also indirectly create internal strain changes in the material being tested, leading to a velocity variation [9]. In this study, an experimental study is conducted in lab conditions with negligible temperature and moisture variations; therefore, the measured sound velocity in the specimen can be viewed as a constant. For general ultrasonic NDT practice, the influence of temperature and load condition should be properly evaluated and compensated. For example, optimal baseline selection [31], baseline signal stretch [32], dynamic time warping [33], and the iterative compensation method [34] have been proposed for temperature compensation for guided wave signals. If temperature variation is uniform, the iterative compensation method can produce a more precise stretch factor. In the case where temperature distribution is uneven, it is suggested that multiple stretch factors are applied to different segments [34].

To evaluate the degree of similarity between the local spectrum *P_i_* and template *T_i_*, an appropriate similarity measure should be designed. A correlation coefficient is widely used as the measure of similarity for template-matching purposes [35]. For the two vectors *P_i_* and *T_i_*, the correlation coefficient between them is calculated by

(12) $$C=\frac{P_{i}\;\times \;T_{i}}{\sqrt{{\left\|P_{i}\right\|}^{2}\cdot {\left\|T_{i}\right\|}^{2}}}=\frac{\langle P_{i},T_{i}\rangle }{\sqrt{\langle T_{i},T_{i}\rangle \cdot \langle P_{i},P_{i}\rangle }}$$

where $\langle T_{i},P_{i}\rangle =T_{i}{}^{T}\cdot P_{i}$ is the inner product of $P_{i}$ and $T_{i}$. The maximum value of C is 1.0 and is obtained when $P_{i}$ and $T_{i}$ are proportional.

It is worthwhile noting that the calculation of correlation coefficient is a linear operation and is appropriate for data that are best described by second-order correlations. In fact, the correlation coefficient is based only on second-order correlations without taking the high-order statistics into account. It is well known that the distribution of the noisy signal is highly irregular and all the “structures” of interest in a spectrum, such as peaks and fluctuations, cannot be described by second-order correlations. This motivates the use of a nonlinear analysis technique that can represent the nonlinear structure of the two spectra. An earlier study showed that by mapping the variables into high-dimensional space before calculating the correlation coefficient, high-order statistics of the variables can be exploited, leading to better detection performance [36].

To better evaluate the degree of similarity between the local spectrum $P_{i}$ and template $T_{i}$, we propose a specially designed similarity measure. First, both $P_{i}$ and $T_{i}$ are nonlinearly mapped into a high-dimensional space F by ${\tilde{P}}_{i}=\Phi (P_{i})$ and ${\tilde{T}}_{i}=\Phi (T_{i})$, respectively, where Φ(⋅) denotes the mapping operation. In this study, the map Φ(⋅) and the space F are determined implicitly by the choice of a kernel function κ, which computes the inner product of mapped $P_{i}$ and $T_{i}$ via:

(13) $$\kappa (P_{i},T_{i})=\langle \Phi (P_{i}),\Phi (T_{i})\rangle$$

It has been proven that if κ is a positive, definite kernel, then a map of Φ(⋅) into an inner product space F exists, such that Equation (13) holds, and the space F has the structure of a so-called Reproducing Kernel Hilbert Space (RKHS) [37]. Using the normalized variables in the feature space, the equation above becomes:

(14) $$\tilde{\kappa }(P_{i},T_{i})=\langle \frac{\Phi (P_{i})}{\left\|\Phi (P_{i})\right\|},\frac{\Phi (T_{i})}{\left\|\Phi (T_{i})\right\|}\rangle =\frac{\kappa (P_{i},T_{i})}{\sqrt{\kappa (P_{i},P_{i})\cdot \kappa (T_{i},T_{i})}}$$

Essentially, Equation (14) is equivalent to calculating the correlation coefficient between $\Phi (P_{i})$ and $\Phi (T_{i})$. In this study, a Gaussian kernel function is used, which has the following form:

(15) $$\kappa (P_{i},T_{i})=\exp(-{\left\|P_{i}-T_{i}\right\|}^{2}/2\sigma^{2})$$

where σ>0 is a parameter that controls the flexibility of the kernel. Note that the denominator in Equation (14) equals 1. It is important to point out that both $P_{i}$ and $T_{i}$ are normalized before evaluating their similarity. Therefore, the similarity between $P_{i}$ and $T_{i}$ can be calculated by:

(16) $$\begin{array}{cc} {\mathrm{MWSS}}_{\left(i\right)} & =\tilde{\kappa }(P_{i},T_{i})\\ & =\exp(-{\left\|P_{i}-T_{i}\right\|}^{2}/2\sigma^{2}) \end{array}$$

This similarity measure above combines two commonly used similarity criteria, the correlation coefficient and the sum of squares of deviations. It can be seen that this similarity measure decays the entries with a low degree of similarity at an exponential scale, such that they are forced to be near zero, and at the same time, preserves the few entries with a high degree of similarity.

The detection of flaws is facilitated by searching the peak values of the MWSS. If MWSS peaks at $t_{j,j=1,2,3,\cdots }$, the locations of flaws can be obtained as $d_{j,j=1,2,3,\cdots }=\frac{1}{2}vt_{j}$, using the time-of-flight principle.

The procedure of the proposed method is summarized as follows:

(1) Measure sound velocity in the material and estimated attenuation coefficient using Equation (11);
(2) Move a time window along the time axis of the measured signal at a fixed step of 1 sample, and the center of the time window is $t_{i}$;
(3) For the time window at $t_{i}$, calculate the spectrum of the windowed data $P_{i}$ by FFT, and construct the template spectrum $T_{i}=\exp[-{\left(f-f_{c}\right)}^{2}/2s^{2}]\cdot \exp[-\frac{1}{2}\alpha {\left(2\pi f\right)}^{4}vt_{i}]$;
(4) Calculate the similarity between $P_{i}$ and $T_{i}$ using Equation (16);
(5) Repeat the above steps until the whole signal is analyzed. Each time window produces 1 entry for MWSSs.
(6) Plot MWSSs verse time. The peaks of MWSSs reveal the time delays of flaw echoes as well as the bottom of the specimen.
(7) Calculate the depths of flaws by the time-of-flight principle.

## 4. Simulation Study

**Figure 2:**
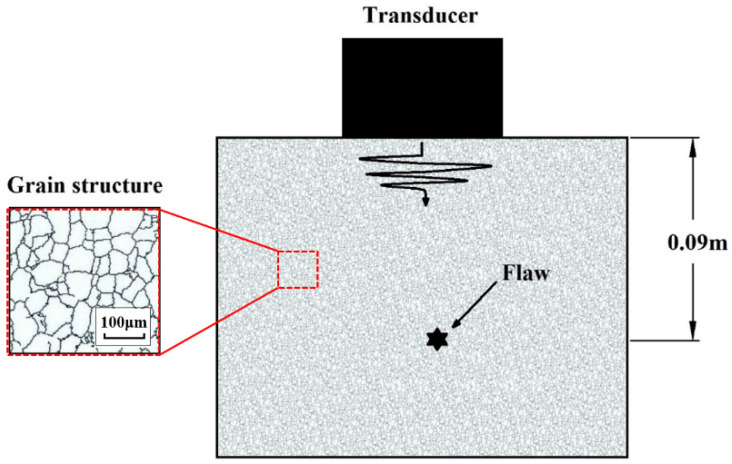
Schematic plot of the NDT scenario for simulation study.

A simulation study was conducted to investigate the performance of the proposed method for a flaw echo signal contaminated with noises. We considered a typical ultrasonic NDT scenario shown in Figure 2, where an ultrasonic transducer with a center frequency of 2 MHz was used to detect a flaw located (indicated by a star) at a depth of 90 mm below the surface. The parameters for generating simulated signals are as follows: attenuation coefficient $\alpha =10^{-28}$, the velocity of the longitudinal wave in the material v=6000 m/s, and the number of grain scatterers K=2500. Therefore, the wavelength in the specimen is λ=3 mm. Comparing wavelength to the grain size, which is assumed to be $\bar{D}=90\;\mu m$, it is seen that $\lambda /\bar{D}\gg 1$; thus, scattering occurs in the Rayleigh region. To generate grain noise, the model proposed in [38] is employed. In this model, the frequency spectrum of grain noise is given by:

(17) $$G(\omega )=H_{t}(\omega )\cdot H_{t}(\omega )\cdot H_{mat}(\omega )$$

where $H_{t}(\omega )$ is modeled as a Gaussian-shaped spectrum and is the frequency response of the transducer, which serves as both transmitter and receiver. $H_{mat}(\omega )$ is the frequency response of the material. ω=2πf is the angular frequency. In the Rayleigh region, $H_{mat}(\omega )$ can be expressed as:

(18) $$H_{mat}\left(\omega \right)=\sum_{k=1}^{K}\beta_{k}\frac{\omega^{2}}{x_{k}}\exp(-2\alpha x_{k}\omega^{4})\exp(-2j\omega x_{k}/v)$$

where α is the attenuation coefficient, $x_{k}$ is the position of the *k*th grain scatterer relative to the transducer and can be modeled as a uniformly distributed random variable such that $x_{k}\in (0,l)$, where l is the thickness of the specimen. K is the number of grains in the ensonified area. Obviously, K is also a random variable, which can be empirically estimated by $K\approx \frac{2l}{\bar{D}}$, with $\bar{D}$ being the mean diameter of grains. The scattering coefficient of the kth grain scatterer $\beta_{k}$ is also a random variable but follows a Rayleigh statistic [39]. In a similar way, the frequency response of a flaw can be expressed as:

(19) $$E(\omega )=H_{t}(\omega )\cdot H_{t}(\omega )\cdot \exp(-2\alpha d_{flaw}\omega^{4})\cdot \exp(-2jd_{flaw}/v)$$

**Figure 3:**
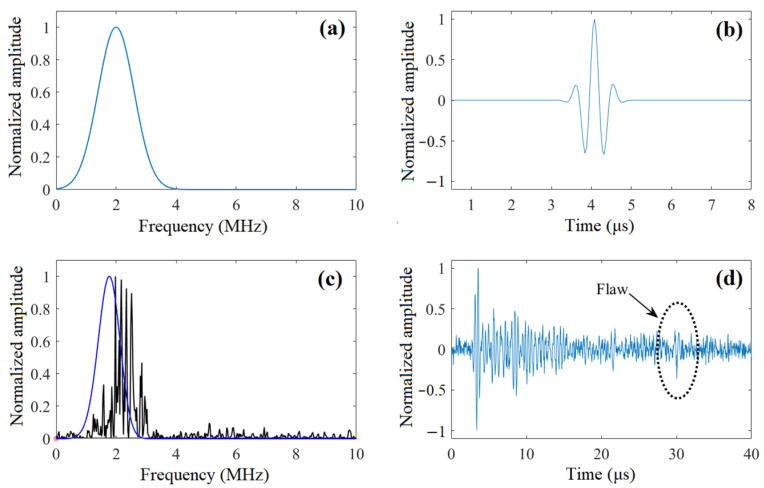
(**a**) Spectrum of the transmitted pulse. (**b**) Waveform of the transmitted pulse. (**c**) The spectra of flaw echo (solid blue line) and grain noise (solid black line). (**d**) The simulated noisy signal; LSNR = 0 dB.

The frequency spectrum and waveform of the transmitted pulse are given in Figure 3a,b, respectively. Based on the model above, the spectra of flaw echo and grain noise are obtained, as shown in Figure 3c. By inverse FFT, the time-domain signal of flaw echo and grain noise can be generated.

To quantitatively characterize noise level, the local signal-to-noise ratio (LSNR) is defined, which is the signal-to-noise ratio within the time window that contains a flaw echo. Suppose the window length is $l_{w}$; the noise is denoted as n(t)=g(t)+w(t), which is the sum of grain noise and white Gaussian noise. The local signal-to-noise ratio can be defined as:

(20) $$\mathrm{LSNR}=10\;\times \;\mathrm{lg}[\sum_{i=p-l_{w}/2}^{p+l_{w}/2}e{\left(i\right)}^{2}/\sum_{i=p-l_{w}/2}^{p+l_{w}/2}n{\left(i\right)}^{2}]$$

The generated time-domain signals contain 1024 samples sampled at a sampling frequency of 25 MHz. After the generation of flaw echo, grain noise, and white Gaussian noise, the three are superimposed to form the noisy signal, and the amplitude of the flaw echo is adjusted to give the designated value of LSNR. In Figure 3d, the simulated noisy signal with LSNR=0 dB is shown, and it is observed that the pattern of grain noise is very similar to the actual flaw echo.

**Figure 4:**
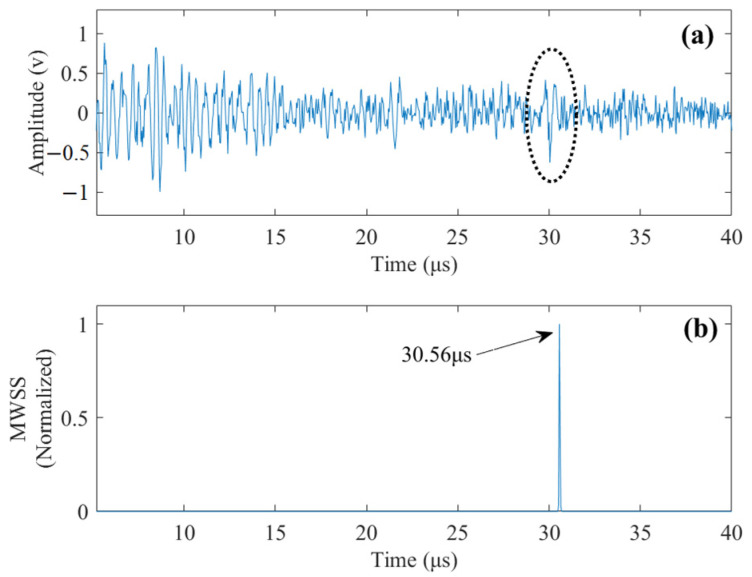
(**a**) Simulated noisy signal, LSNR = 0 dB. (**b**) The MWSSs by the proposed method.

By implementing the proposed method, the obtained MWSS is demonstrated in Figure 4. The peak of MWSS occurs at *t_f_* = 30.56 μs, revealing a depth of flaw of 91.7 mm. Compared with the actual flaw depth of 90 mm, the detection result is very accurate, with a relative error of just 1.9%.

**Figure 5:**
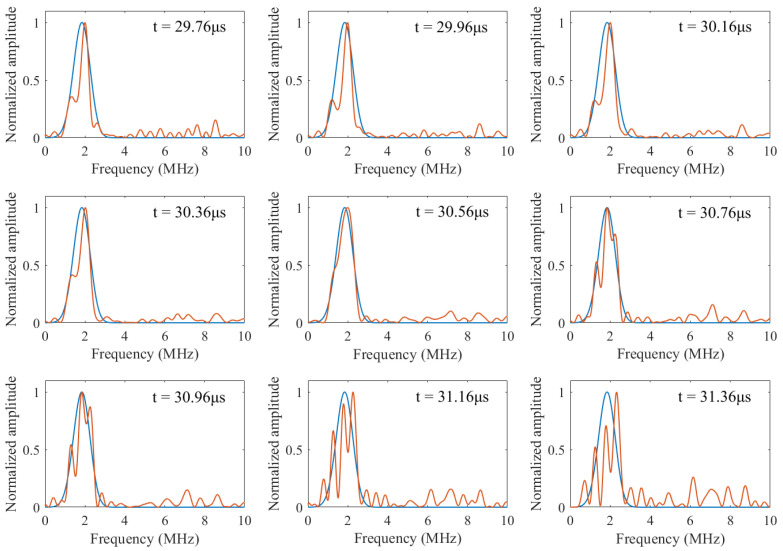
Spectra of time windows adjacent to *i* = 30.56 μs along with their corresponding template spectra (solid red line: spectrum of windowed data; solid blue line: template spectrum).

In Figure 5, spectra of the time windows adjacent to the peak of MWSS are shown along with their corresponding template spectra. It can be observed that at *t* = 30.56 μs, the spectrum of windowed data is very close to its template spectrum, and all the time windows away from *t* = 30.56 μs show significant differences between the spectra of windowed data and template spectra. Notice that the sum of squares of deviations (SSD) between *P_i_* and *T_i_* is the norm term in Equation (16); therefore, the MWSSs for these time windows will be much lower than the MWSS at *t* = 30.56 μs. The normalized MWSS values for the time windows in Figure 5 are tabulated in Table 1. Most of the MWSS values are close to zero, which can also be observed in Figure 4b.

## 5. Experimental Studies

**Figure 6:**
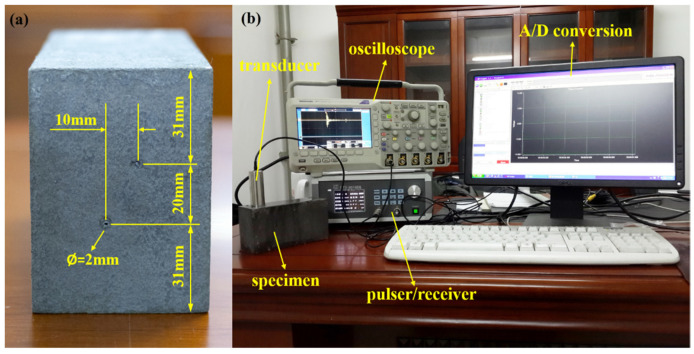
(**a**) Heat-treated stain steel specimen with two flaws. (**b**) The setup of the experiment system.

To further evaluate the performance of the proposed method for flaw detection, we conduct a flaw-detection experiment shown in Figure 6. The specimen under inspection is a heat-treated type 304 stainless steel block with a cross-section dimension of 82 mm × 59 mm (height by width, respectively). The heat treatment is implemented at a temperature of 1250 °C for 8 h, followed by quenching in a water bath. Empirically, grain sizes $\bar{D}\approx 120\;\mu m$ will be formed. Two side-drilled 2 mm-diameter holes are considered as flaws, and they are located about 31 mm and 51 mm below the top surface, respectively, as shown in Figure 6a. A transducer with a center frequency of 5 MHz (type 5P20 made by Shantou Institute of Ultrasonic Instruments Co., Ltd., Shantou, China) is used. A-scan signals are digitized by an A/D converter (ADVANTECH PCI-9820) and sampled at a sampling frequency of 60 MHz. The experimental setup is demonstrated in Figure 6b.

Sound velocity is measured first. The measurement of sound velocity is based on the time-of-flight principle. The thickness of the specimen is measured ten times using a Vernier caliper and the results are averaged, yielding the thickness of the specimen l=81.4 mm. The round-trip travel time of the ultrasonic pulse reflected by the bottom of the specimen is 28.4 μs; therefore, the sound velocity equals v=5735 m/s. Up until now, one can notice that the wavelength in the specimen is λ≈1 mm; thus, $\lambda /\bar{D}\gg 1$, so the scattering is in the Rayleigh region.

**Figure 7:**
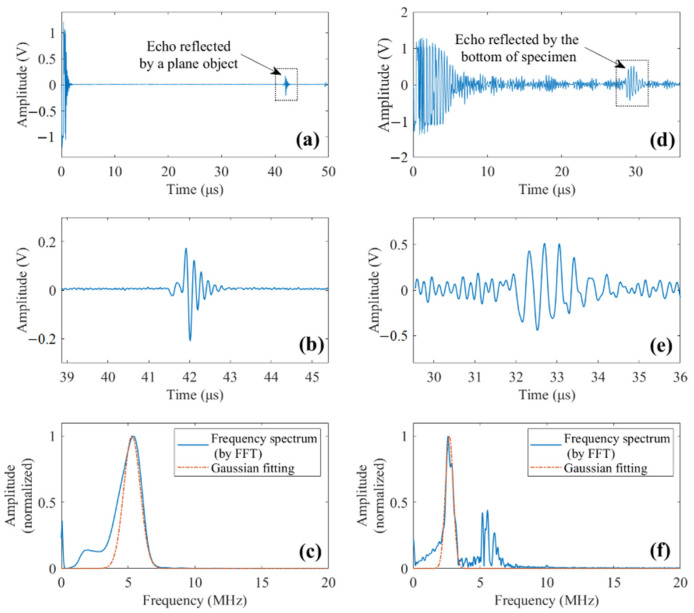
(**a**) Plane-reflected waveform. (**b**) The pulse reflected by a plane object. (**c**) Original and Gaussian-fitted frequency spectrum of the plane-reflected pulse. (**d**) Waveform of an experimental signal. (**e**) Bottom-reflected echo. (**f**) Original and Gaussian-fitted frequency spectrum of the bottom-reflected echo.

To estimate the attenuation coefficient, the transmitted pulse from the transducer and the bottom-reflected echo should be obtained first. In practice, the former can be obtained by positioning the transducer normally to a plane reflection in the far field and measuring the reflected signal. In this study, the plane-reflected waveform is shown in Figure 7a, where the reflected pulse is highlighted and shown in Figure 7b. The frequency spectrum of this pulse is obtained by FFT and given in Figure 7c. To better measure the center frequency, a Gaussian function is employed to fit the calculated spectrum, and the fitting result is also shown in Figure 7c. The estimated center frequency of the transmitted pulse is $f_{c}=5.27\;\mathrm{mHz}$. A signal obtained from the NDT experiment is presented in Figure 7d, from which the bottom-reflected pulse can be identified in the highlighted region. The waveform and frequency spectrum of the bottom-reflected pulse are shown in Figure 7e,f, respectively. The center frequency of the bottom-reflected pulse is $f_{b}=2.70\;\mathrm{mHz}$. Substituting $f_{c}$ and $f_{b}$ into Equation (11) yields an attenuation coefficient $\alpha =3.41\;\times \;10^{-28}$.

**Figure 8:**
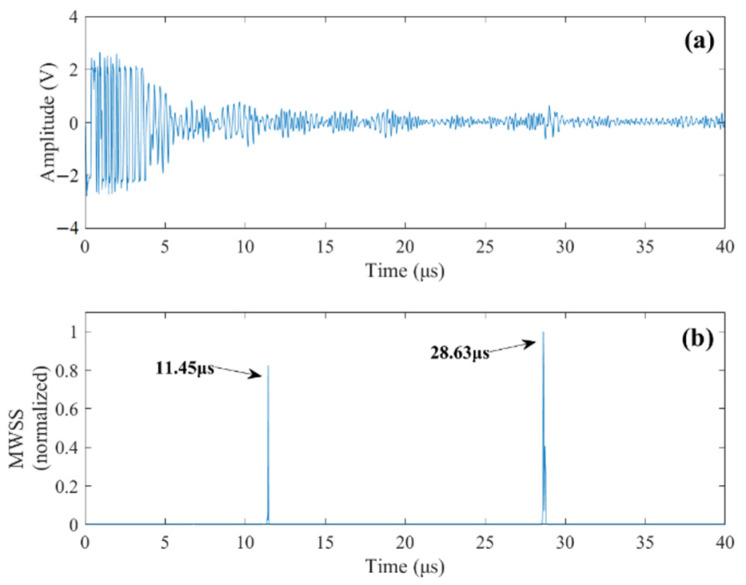
(**a**) A-scan signal of the upper flaw. (**b**) MWSS values revealing the time delays of the upper flaw echo and bottom reflection.

Case 1: Single flaw detection. In this case, the transducer is positioned right above the upper flaw. The measured signal from the testing is presented in Figure 8a. Strong grain noise can be observed from the measured signal. Implementing the proposed method, the detection results are shown in Figure 8b. The obtained MWSSs clearly reveal the upper flaw as well as the bottom of the specimen. The detected depths of the upper flaw and bottom of the specimen are given in Table 2. Comparing the detection results with their actual values, it is observed that high detection accuracy can be obtained by the proposed method.

**Figure 9:**
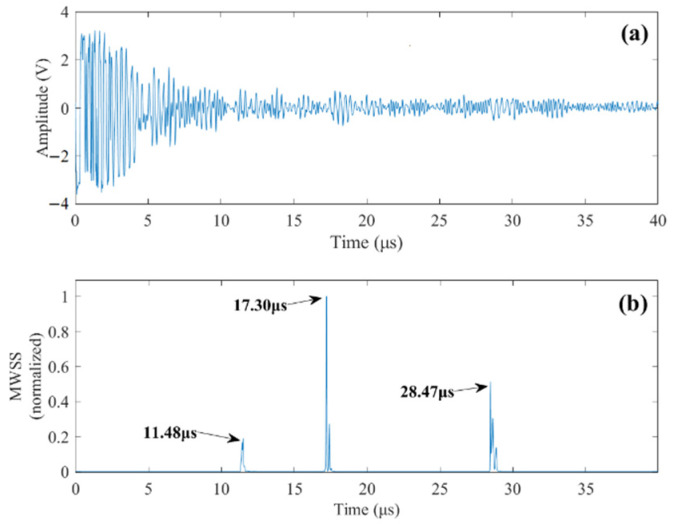
(**a**) A-scan signal of both upper and lower flaws. (**b**) Obtained MWSS values revealing the time delays of the two flaw echoes and bottom reflection.

Case 2: Simultaneous detection of two flaws. In Figure 9a, an experimental signal obtained when the transducer is positioned between the two flaws so the received signal contains two flaw echoes is presented. Using the proposed method, the resulting MWSSs are shown in Figure 9b, from which three peaks are clearly identified, revealing time delays corresponding to the upper flaw, the lower flaw, and bottom of the specimen, respectively. It can be observed from the experimental signal that the echo reflected by the lower flaw has a higher LSNR than the upper flaw; therefore, the MWSS value for the lower flaw is higher. Although the flaws are indistinguishable from the time-domain signal, they can be successfully identified by the proposed method.

Using the time-of-flight principle, the depths of the flaws are obtained and compared with their actual values. The results are tabulated in Table 3, showing satisfactory accuracy of the proposed method.

## 6. Conclusions

In this work, a novel approach is proposed for flaw detection in highly scattering materials using a simple ultrasonic sensor. The feature that the spectrum of a flaw echo shows a different pattern compared to that of grain noise at a given time is exploited, facilitating the detection of flaws by an adaptive template-matching scheme. Moreover, a new method for estimating the frequency-dependent attenuation coefficient, which is easy to use in practice, is also developed. This approach has been demonstrated both in a simulation study and experimental studies and in both cases, the flaw-detection accuracy is high, showing the good performance of the proposed method.

It should be noted that although the main aim in this work is to develop an efficient method to detect flaws in applications where grain noise is present, it is safe to conclude that the proposed method works even better if the noise is white Gaussian from the fact that spectrum of white Gaussian noise shows a more pronounced difference from that of a flaw echo. It has shown the capability for effective flaw detection and potential for applications in the field, once the effects of changing environmental and operational conditions such as temperature variations and load conditions, etc., are fully taken into account, yet mitigation against such effects remains an open problem.

## Figures and Tables

**Table 1:** Normalized MWSSs for the time windows shown in Figure 5.

Time Window	29.76 μs	29.96 μs	30.16 μs	30.36 μs	30.56 μs
MWSS (normalized)	$1.2\;\times \;10^{-85}$	$1.3\;\times \;10^{-87}$	$4.8\;\times \;10^{-65}$	$5.1\;\times \;10^{-46}$	1
Time window	30.76 μs	30.96 μs	31.16 μs	31.36 μs	
MWSS (normalized)	$1.3\;\times \;10^{-40}$	$5.9\;\times \;10^{-56}$	$4.4\;\times \;10^{-158}$	$1.0\;\times \;10^{-268}$	

**Table 2:** Detection results of the proposed method for case 1.

	Upper Flaw	Bottom
Detected depth (mm)	32.9	82.1
Actual depth (mm)	31.0	81.4
Absolute error (mm)	1.9	0.7

**Table 3:** Detection results of the proposed method for case 2.

	Upper Flaw	Lower Flaw	Bottom
Detected depth (mm)	32.9	49.6	81.6
Actual depth (mm)	31.0	51.0	81.4
Absolute error (mm)	1.9	1.4	0.2

## Data Availability

Not applicable.
